# Implantation of autologous muscle-derived stem cells in treatment of fecal incontinence: results of an experimental pilot study

**DOI:** 10.1007/s10151-015-1351-0

**Published:** 2015-08-13

**Authors:** M. Romaniszyn, N. Rozwadowska, A. Malcher, T. Kolanowski, P. Walega, M. Kurpisz

**Affiliations:** 3rd Department of General Surgery, Jagiellonian University Medical College, ul. Pradnicka 35-37, 31-202 Kraków, Poland; Department of Reproductive Biology and Stem Cells, Institute of Human Genetics, Polish Academy of Science, Strzeszynska 32, 60-479 Poznan, Poland

**Keywords:** Fecal incontinence, Electromyography, Anal manometry, Stem cells, Myoblasts, Muscle regeneration

## Abstract

**Background:**

The aim of this study is to present results of the implantation of autologous myoblasts into the external anal sphincter (EAS) in ten patients with fecal incontinence.

**Methods:**

After anatomical and functional assessment of the patients’ EAS, a vastus lateralis muscle open biopsy was performed. Stem cells were extracted from the biopsy specimens and cultured in vitro. Cell suspensions were then administered to the EAS. Patients were scheduled for follow-up visits in 6-week intervals. Total follow-up was 12 months.

**Results:**

All biopsy and cell implantation procedures were performed without complications. Nine of the patients completed a full 12-month follow-up. There was subjective improvement in six patients (66.7 %). In manometric examinations 18 weeks after implantation, squeeze anal pressures and high-pressure zone length increased in all patients, with particularly significant sphincter function recovery in five patients (55.6 %). Electromyographic (EMG) examination showed an increase in signal amplitude in all patients, detecting elevated numbers of propagating action potentials. Twelve months after implantation two patients experienced deterioration of continence, which was also reflected in the deterioration of manometric and EMG parameters. The remaining four patients (44.4 %) still described their continence as better than before implantation and retained satisfactory functional examination parameters.

**Conclusions:**

Implantation of autologous myoblasts gives good short-term results not only in a subjective assessment, but also in objective functional tests. It seems that this promising technology can improve the quality of life of patients with fecal incontinence, but further study is required to achieve better and more persistent results.

## Introduction

The incidence of fecal incontinence in the general population is estimated to be 2–15 %, increasing with age. It drastically reduces quality of life and leads to reduction in social activity and withdrawal from social and professional life. Moreover, it leads to severe depression and anxiety [1]. The healthcare costs associated with this disease are enormous. The estimated cost of one patient treatment with fecal incontinence exceeds $4000 per year [2].

The most common pathological mechanism of fecal incontinence is the insufficiency of the external anal sphincter (EAS) caused by neurological or myogenic dysfunction. The myogenic mechanism of EAS insufficiency is usually due to direct mechanical damage during childbirth, trauma or surgery in the anorectal region, whereas the neurological mechanism involves damage to either the spinal or peripheral nerves, in most cases the pudendal nerve. Unfortunately, coincidence of sphincter rupture with damage to pudendal nerves is quite common [3].

There are many methods for treating fecal incontinence; however, the efficacy of each of them is limited [4–10]. Conservative management, dietary and pharmacological treatment and biofeedback training techniques give satisfactory results only in patients with lower grades of incontinence [5–7]. As for surgical methods, only some patients achieve long-term satisfactory results. Overlapping sphincteroplasty, a classical surgical treatment for sphincter damage, improves the clinical status of patients, but the benefit is maintained only in half of the patients in long-term follow-up [8–10].

Each skeletal muscle, including the EAS, has the ability to regenerate (to some degree) and to repair damage sustained. A special stem cells, called satellite cells, located among the muscle fibers, are responsible for this phenomenon. In response to injury and/or muscle damage, satellite cells are activated and become myoblasts—capable of intense proliferation. Myoblasts then differentiate and fuse together to form new muscle fibers and connect with existing ones, adding new portions of contractile tissue to existing motor units [11].

Isolated satellite cells in an undifferentiated state are relatively easy to culture in vitro, and after implantation they only differentiate into muscle cell lines, regardless of the type of tissues in the vicinity, do not proliferate more than necessary to repair the damaged muscle and show no tendency toward malignant transformation. Additionally, the use of autologous myoblasts is a way to avoid problems related to possible immune rejection or necessity of immunosuppression [12]. The technology of in vitro myoblast culturing is being constantly improved and is well documented in the scientific literature [12–15].

Attempts at auto-transplantation of myoblasts into damaged skeletal muscle have already been made in animal models of muscular dystrophy [16], postinfarction myocardial dysfunction [12, 17–19] and urethral sphincter insufficiency [20]. Another example of effective cell therapy using autologous myoblasts is the treatment of stress urinary incontinence [21, 22]. The mechanism of this disorder very often involves failure of the external urethral sphincter, with similar etiology as in fecal incontinence.

Some attempts have already been made to use autologous myoblast injections to augment the EASs, mostly on animal models, but also on small groups of selected patients [23, 24]. Based on those encouraging results, a pioneer experimental study was designed in attempt to enhance the function of the EAS using injections of autologous muscle-derived stem cells, the myoblasts.

## Materials and methods

The study was designed as a pilot prospective experimental clinical trial. It was conducted by two cooperating facilities—the 3rd Department of General Surgery, Jagiellonian University in Krakow and the Department of Reproductive Biology and Stem Cells, Institute of Human Genetics of the Polish Academy of Sciences in Poznan. The study design was approved by the Jagiellonian University Bioethics Committee.

Patients with fecal incontinence due to sphincter insufficiency of various origin, who failed initial 6-month biofeedback, were offered all appropriate treatment options available in the 3rd Department of General Surgery, Jagiellonian University, including stem-cell-based treatment. Ten patients, who chose to undergo the autologous myoblast implantation, were enrolled in this study. Written informed consent was obtained from all patients.

Inclusion criteria include the following:

- age 18–75 years;
- moderate-to-severe fecal incontinence (Wexner score >10);
- reduced basal and squeeze pressure on anorectal manometry, with preserved anorectal reflexes [recto-anal inhibitory reflex (RAIR) and rectal sphincter contraction on cough (RSCC)] and anorectal sensation;
- preserved sphincter innervation based on properly propagating action potentials of motor units shown in sphincter electromyography;
- informed consent of the patient.

Exclusion criteria include the following:

- Fourth degree fecal incontinence (Wexner score = 20);
- EAS defect larger than 1/4 of sphincter circumference;
- EAS deinnervation, sensory deinnervation (neurogenic etiology);
- advanced age (>75 years old);
- concomitant colorectal disorders [including rectocele, intussusception, rectal prolapse, advanced hemorrhoidal disease (third–fourth degree)] or musculoskeletal disorders including lower extremity ischemia;
- other health disorders which could potentially increase the risk of planned procedures (severe heart, lung, hepatic or renal disorders, severe diabetes).

**Fig. 1:**
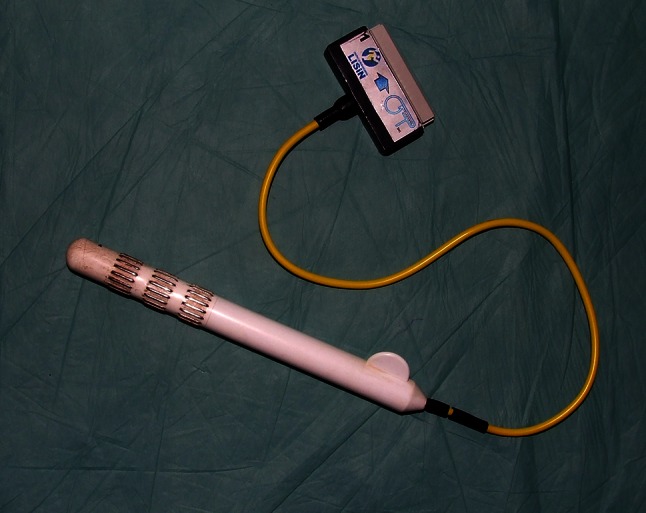
Surface electromyography rectal probe

**Fig. 2:**
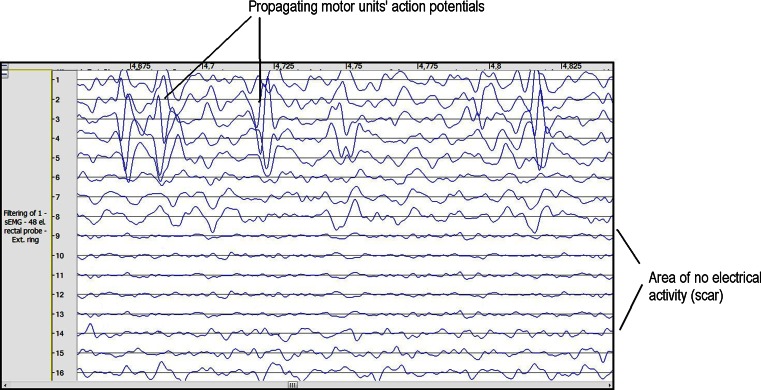
Example of electromyography before implantation
- Endorectal ultrasound (ERUS). The external sphincter muscle was assessed visually, with measurements of muscle thickness and dimensions of any scarring or defects present.

A total of one male and nine female patients with a mean age of 43 years (range 20–68 years) were enrolled in the study. The enrolled patients suffered from fecal incontinence of varying degree and etiology (six with EAS damage and four with idiopathic fecal incontinence). Apart from subjective symptom assessments (Wexner and Fecal Incontinence Severity Index (FISI) questionnaires), a series of examinations were performed:

- Anorectal manometry (Medtronic™ Polygraph^®^ 4-Channel Air-Charged Manometer). The following parameters were recorded: mean resting pressure (BAP), squeeze pressure (SAP) and high-pressure zone length (HPZL).
- Surface endoanal electromyography (OTBioElettronica, Turin, Italy). A 48-channel 3-ring endoanal probe (Fig. 1) was used to obtain repetitive 10-s acquisitions during rest and maximum contraction of the EAS. The signal was analyzed visually for propagating action potentials, and the following parameters were recorded: mean root square of amplitude of motor unit action potentials (ARV), mean signal frequency (MNF) and median signal frequency (MDF). Also, dimensional maps of signal strength were generated (Fig. 2).

Under local anesthesia, a small incision of about 3 cm was made on lower lateral side of the thigh, over the vastus lateralis muscle (lateral head of the quadriceps of the thigh). After preparation of tissues, the epimysium was dissected and a 1-cm sample of muscle was harvested. After careful hemostasis, the wound was closed with surgical sutures. The specimens were sent to the Department of Reproductive Biology and Stem Cells for in vitro culturing of the myoblast cells.

The tissue fragment was mechanically dissected and subjected to digestion with 0.02 % collagenase solution (Sigma). The obtained cell suspension was filtered trough 80-µm mesh, centrifuged and plated on gelatine-covered culture dish. Unless stated otherwise, Lonza supplied all reagents used for in vitro culture media preparation. The cells were kept in a 5 % CO_2_ atmosphere in 37 °C in 95 % humidity. Dulbecco’s modified Eagle’s medium (DMEM) was supplemented with 20 % fetal bovine serum (FBS), Ultraglutamine^®^, antibiotic and antimycotic agents. To facilitate the optimal proliferation environment and to inhibit the spontaneous myocytes formation, the basic fibroblast growth factor (bFGF) was provided (individually tailored concentrations). The myoblasts were cultured up to 70 % confluence to avoid spontaneous cell fusion and passaged every 4–5 days (altogether 7–10 passages), while the medium was replaced every other day. The myoblasts were mostly cultured for approximately 5.5 ± 0.8 weeks. Efficiency of cell culture was evaluated after the final passage of cells indicating the number of CD56 positive cells as well as their myogenic characteristics by range of gene expression in reverse transcription polymerase chain reaction (RT-PCR). Also, the cell suspensions were tested in routine microbial smears including mycoplasms. The final number of cultured cells ranged from 50 to 600 × 10^6^ (with a mean of 249 ± 68 × 10^6^) from 1 cm of isolated muscle tissue fragment.

To confirm myogenic properties of obtained cells, their differentiation potential was assessed. Myocyte in vitro formation was induced after cells reached 90 % confluence. The differentiation medium (DM) was supplemented by 2 % horse serum instead of 20 % FBS and was deprived of growth factors.

The CD56 expression [surface marker of neural, glial, natural killer (NK) and also myogenic cells] was evaluated using flow cytometry. Cells were stained with PE-Cy5™ (PC5)-conjugated anti-CD56 antibody according to manufacturer’s protocol (Beckmann Coulter, Brea, CA, USA). The cells were resuspended in 20 % FBS [in phosphate-buffered saline (PBS)] and incubated with PC5-conjugated anti-CD56 antibody for 20 min. After adding 2 % FBS (diluted with PBS), the cells were centrifuged in 1200 rpm for 5 min and the subsequent pellet was washed with 2 % FBS solution (in PBS) and again centrifuged. Finally, the cells were resuspended in 20 % FBS solution and 104 cells were analyzed in the Cell Lab Quanta Cytometer (Beckmann Coulter, Brea, CA, USA).

Total ribonucleic acid (RNA) was extracted from 2 × 10^6^ of cells according to the manufacturer’s protocol of Tri^®^reagent (Sigma-Aldrich, St. Louis, MO, USA). Ten micrograms of total RNA was used to purify messenger (m) RNA fraction using Dynabeads^®^ mRNA Purification Kit (Invitrogen Life Technologies, Carlsbad, CA, USA). The reverse transcription reactions were performed in standard conditions using Superscript reverse transcriptase III (Invitrogen Life Technologies, Carlsbad, CA, USA). The real-time PCR was performed using iQ SYBR Green Supermix (Bio-Rad, Hercules, CA, USA).

The threshold cycle (*C*_t_) values of each studied transcript were analyzed with iCycler iQ5 Real-Time PCR Detection System (Bio-Rad Laboratories). All samples and standard curve were run in duplicate. The relative expression level of each studied transcript (DES, MYOD1, MYOG, MHC1, MRF4, MYF5) was normalized with reference to three housekeeping genes (ACTB, TBP, and GAPDH) according to Vandesompele et al. [25]. Primer sequences are summarized in Table 1.

**Table 1:** Primer sequences for all target genes used in real-time polymerase chain reaction

Gene ID	Name of gene	Primers	Primer sequences (5′ → 3′)	Product size (bp)
*ACTB*	β-Actin	Forward	CTTCCTGGGCATGGAGTCC	192
		Reverse	ATCTTGATCTTCATTGTGCTG	
*DES*	Desmin	Forward	CAGGTGGAGGTGCTCACTAAC	123
		Reverse	TGTTCTCTGCTTCTTCCTTCAAC	
*GAPDH*	Glyceraldehyde-3-phosphate dehydrogenase	Forward	GCTCTCTGCTCCTCCTGTTC	112
		Reverse	ACCAAATCCGTTGACTCCGA	
*MHC1*	Myosin heavy chain 1	Forward	CCCTTGAGAAGACGAAGCAGAG	193
		Reverse	GTGAGCGGGATTCCTTTTGAG	
*MYF5*	Myogenic factor 5	Forward	TGCAGGAGTTGCTGAGAGAGCA	120
		Reverse	CAGGACTGTTACATTCGG	
*MYOD1*	Myogenic differentiation 1	Forward	ACGGCATGATGGACTACAG	212
		Reverse	CGACTCAGAAGGCACGTC	
*MYOG*	Myogenin	Forward	GCTGTATGAGACATCCCCCTA	226
		Reverse	CGACTTCCTCTTACACACCTTAC	
*MRF4*	Myogenic regulatory factor 4	Forward	CTTCAGCTACAGACCCAAACA	94
		Reverse	CCTGGAATGATCGGAAACAC	
*TBP*	TATA box binding protein	Forward	CATGACCCCCATCACTCCTG	196

The cell population before transplantation was analyzed using Annexin V-FITC test. This assay evaluates the number of apoptotic [with outer membrane-exposed phospholipid phosphatidylserine (PS)] cells. Necrotic cells were labeled with propidium iodide.

Cellular senescence is defined as growth arrest and cell cycle exit. The myoblast population was kept over 5 weeks in in vitro conditions, so there was a need to establish the number of cells in sample population that reached their proliferation limit. To analyze this process, the expression of SA-beta-galactosidase was followed with the using of X-Gal substrate. A staining kit based on histochemical reaction was used (Senescence Detection Kit—Biovision) according to manufacturer manual. Briefly, cells were fixed and left overnight wit X-Gal staining solution in 37 °C. After 24 h, the blue color was developed in cells expressing SA-beta-galactosidase and the specimens were counterstained with eosin to visualize the cytoplasm of all cells.

After preparation of the site, local anesthesia was administered (1 % lidocaine s.c.) and a suspension of 10^8^ myogenic stem cells per ml in DMEM with 1 % human albumin was administered deep into the EAS under ERUS guidance. Total volume of the suspension (about 3 ml) was injected on entire circumference of the external sphincter muscle ring (several injections about 1 cm apart). In patients with a confirmed EAS defect, the total volume of the suspension was divided into three 1-ml portions: 1 ml was injected on both sides of the muscle scar, one was applied on the remaining circumference of external sphincter muscle ring (injections about 1 cm apart), and the last portion was injected directly into the scar, so that the bolus of injected fluid had direct contact with healthy muscle tissue, creating a “bridge” between muscle ends, across the scar. Patients went home the same day.

The patients were scheduled for 4 follow-up visits: at 6, 12, 18 weeks and 12 months after implantation. On each of the follow-up visits, a series of examinations was performed: anorectal manometry, surface endorectal EMG, ERUS and questionnaire assessments.

The data were analyzed using StatSoft STATISTICA^®^ software. Mann–Whitney *U* and Friedman ANOVA tests were used to assess differences between measurements, and Spearman correlation test was used for correlation assessments.

## Results

In the initial study group assessment, mean BAP was 34 mmHg, mean SAP was 63 mmHg, mean HPZL was 14.8 mm, while the mean Wexner score was 15 (range 12–18) and the mean FISI score was 36 (range 29–45). EMG assessment showed the presence of motor unit action potentials in all patients, with visible gaps in the six patients with EAS defects, confirmed by means of ERUS.

**Fig. 3:**
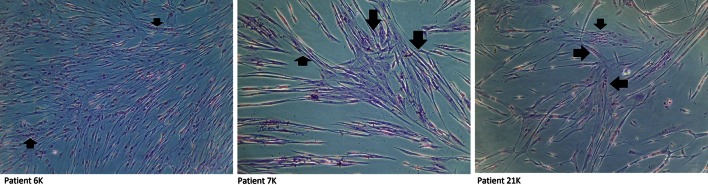
Representative micrographs indicate positive differentiation of patients’ myoblasts into myotubes. *Black arrows* indicate the multinucleated cells under differentiation conditions

Tissue harvesting was performed under local anesthesia and patients were discharged home the same day. Postoperative pain was easily controlled with oral paracetamol, and none of the patients found the procedure inconvenient. There were no complications of this procedure.

Implantation of the myoblast suspension was also performed under local anesthesia, under direct ultrasound guidance. There were no complications. One of the patients reported mild discomfort in the perianal region, which persisted a few hours after injection. The complaint resolved spontaneously without further implications.

**Fig. 4:**
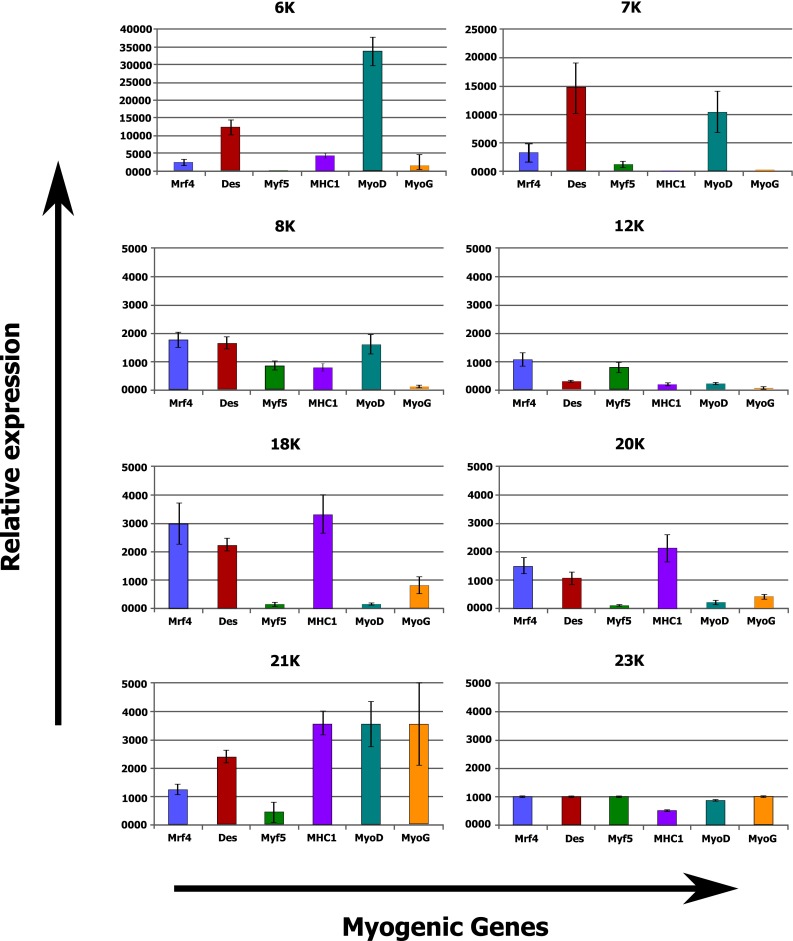
Examples of relative expression of myogenic genes for individual patients (6K–23K: patients’ unique identifiers)

The cells obtained showed typical myoblast morphology with in vitro myocyte formation evidenced by multinuclear myotube presence (Fig. 3) and high proliferative potential. After an average of 5.5 ± 0.8 weeks of in vitro culture, from 50 to 600 × 10^6^ cells (with a mean of 249 ± 68 × 10^6^ from 1 cm of isolated muscle tissue fragment) were prepared for the transplantation procedure. A majority of cells presented surface CD56 marker; however, three different subgroups of patients could be observed, those with a high CD56 expression (87 ± 4 % cells), moderate expression (at the level of 60 ± 8 %) and a low proportion of CD56 positive cells (below 50 %). We also performed the validation of selected myogenic markers to analyze cell population characteristics. We observed that the majority of examined cell populations presented a medium intensity of desmin, whereas the MYOD1 and MYOG protein expression showed rather heterogenous and weak intensity (data unpublished). Thus, the myoblast population was mostly determined as the proportion positive for CD56 marker, and there were also myogenic genes expression by real-time PCR. We observed that each cell population obtained was of myogenic origin, but at a different stage of cell differentiation, which suggests that culture conditions were individual for each patient (Fig. 4).

The myoblast populations showed on average 16.94 ± 10.41 % of necrotic cells, whereas cells with apoptotic characteristics stood for 1.87 ± 0.89 % of population. The evaluation of SA-beta-galactosidase expression showed that more than half of the cell population (55.94 ± 24.82 %) besides a relatively long in vitro culture retained their “young” characteristic. The presence of a moderate level of beta-galactosidase positive cells was observed in 39.84 ± 21.67 % of the cell population, while only 4.22 ± 3.47 % of cells showed high expression of the senescence indicator. At least 12 % of cells was defined as an actively proliferating population at cell transplantation time-point (6.04 ± 3.64 in S phase and 6.11 ± 3.93 in G2 phase). All cell samples were capable of an efficient differentiation process—fusion index (percentage of nuclei incorporated in myotubes) estimated at 0.62 ± 0.18 %. There was a significant negative correlation between the patient’s age and number of cultured cells (Spearman *R* = −0.7), which also needed significantly more time in some cases to reach readiness for implantation.

**Fig. 5:**
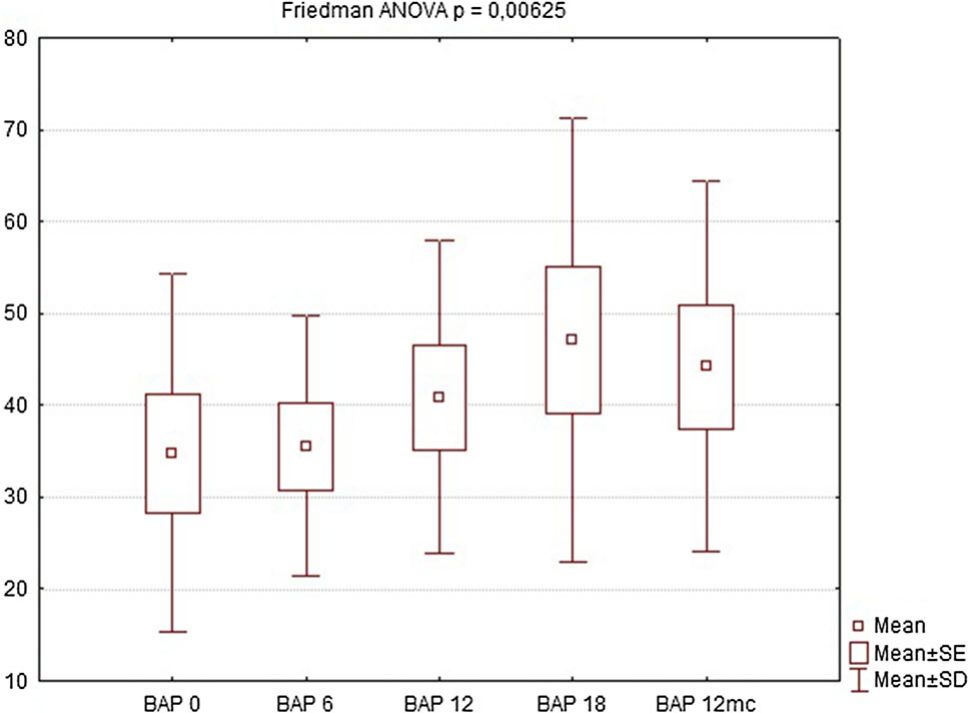
Resting pressure (BAP) in cmH_2_O initially and after 6, 12, 18 weeks and 12 months

**Fig. 6:**
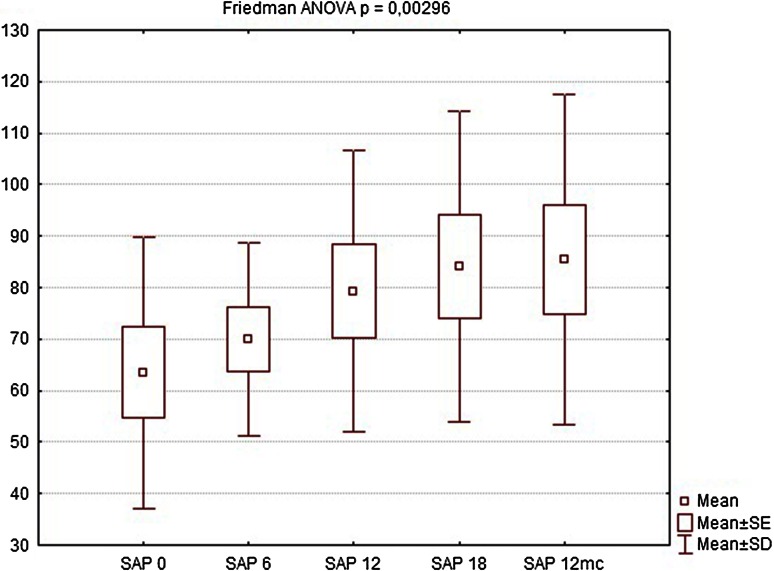
Squeeze pressure (SAP) in cmH_2_O initially and after 6, 12, 18 weeks and 12 months

**Fig. 7:**
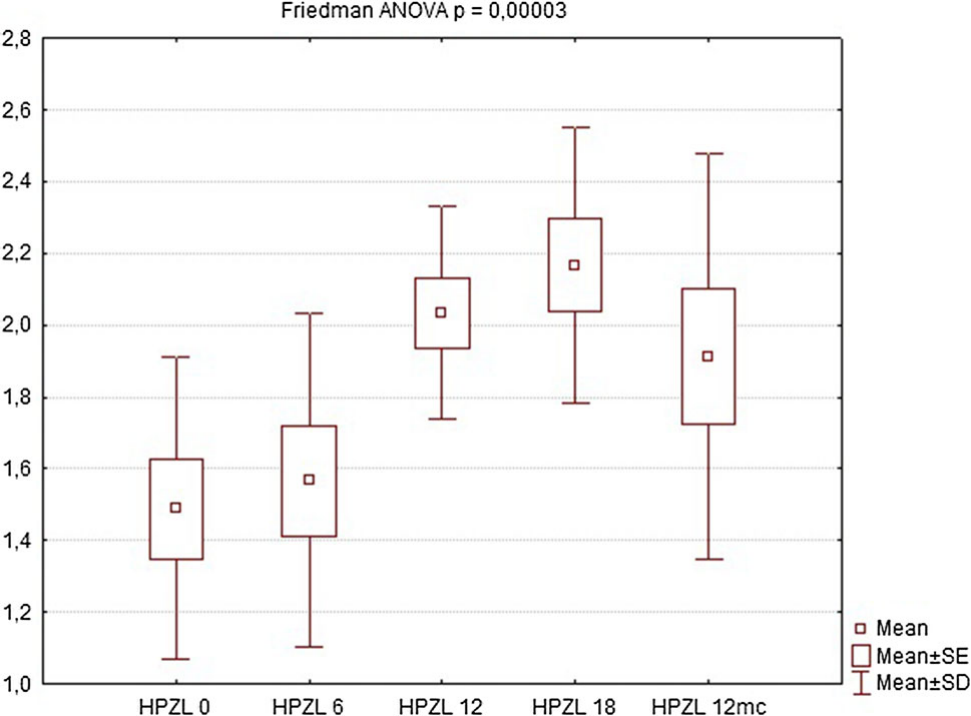
High-pressure zone length (HPZL) in cm initially and after 6, 12, 18 weeks and 12 months

**Fig. 8:**
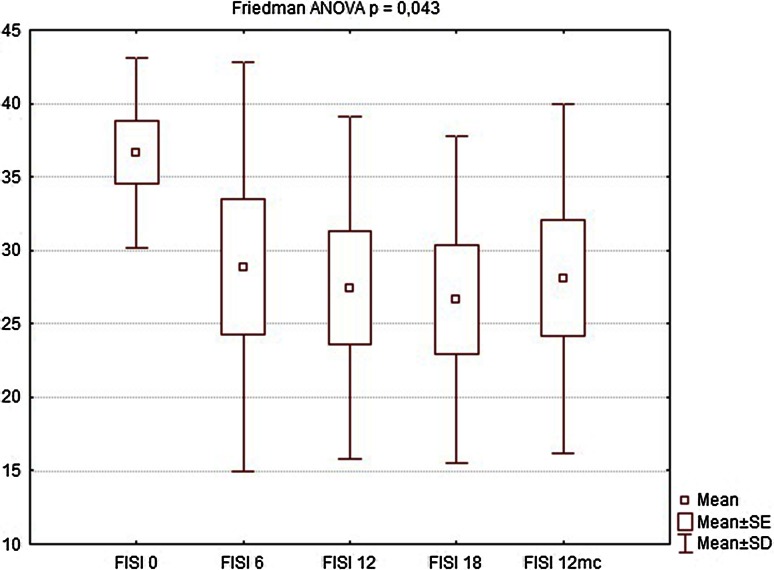
Fecal Incontinence Severity Index (FISI) score—initially and after 6, 12, 18 weeks and 12 months

**Fig. 9:**
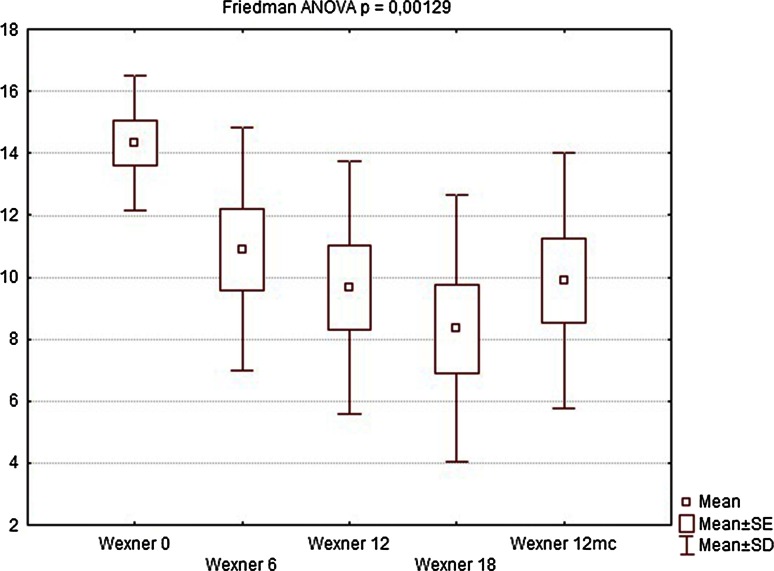
Wexner score—initially and after 6, 12, 18 weeks and 12 months

Nine of the patients completed the full 12-month follow-up (one patient refused to undergo further examinations and was considered lost to follow-up). During the first follow-up visit after 6 weeks, there were no significant changes in manometric examinations regarding all parameters, BAP, squeeze pressure SAP, and HPZL, although some patients reported subjective improvement in questionnaire evaluation. The site of implantation showed no signs of edema, nor was there any significant difference in ultrasound assessment. At the following visits (12 and 18 weeks after implantation), SAP and HPZL were gradually increasing (Figs. 5, 6, 7), as scores in incontinence questionnaires were decreasing (Figs. 8, 9). There was also a slight increase in BAP. After 18 weeks of follow-up, SAP was significantly increased in most patients (on average by 20.5 mmHg), with a mean relative increase 38.3 % of the initial pressures (Friedman *p* < 0.05). HPZL was significantly longer (average increase of 6.7 mm), and the mean relative increase was 51.7 % of the initial values (Friedman *p* < 0.05). The increase in BAP, although less intense, also reached statistical significance. Mean values of the measurements are presented in Table 2.

**Table 2:** Comparison of manometry (BAP, SAP, HPZL), questionnaire (FISI, Wexner) and EMG values before implantation and 6, 12, 18 weeks and 12 months after implantation. Results are mean values

	Initial	6 weeks	12 weeks	18 weeks	12 months
BAP (cmH_2_O)	34.78	35.56	40.89	47.11	44.22
SAP (cmH_2_O)	63.56	70.00	79.33	84.11	85.44
HPZL (cm)	1.49	1.57	2.03	2.17	1.91
FISI	36.67	28.89	27.44	26.67	28.11
Wexner	14.33	10.89	9.67	8.33	9.89
EMG amplitude (µV)	5.80	7.43	8.63	9.88	8.50
EMG frequency (Hz)	96.78	91.81	88.24	85.94	91.00

*BAP* resting anal pressure, *SAP* squeeze pressure, *HPZL* high-pressure zone length, *FISI* Fecal Incontinence Severity Index, *EMG* electromyography

In a case-by-case analysis after 18 weeks, significant subjective improvement was obtained in six patients (66.7 %). In manometric examinations, SAP and HPZL increased in all patients, with a particularly significant increase in five patients (55.6 %).

**Fig. 10:**
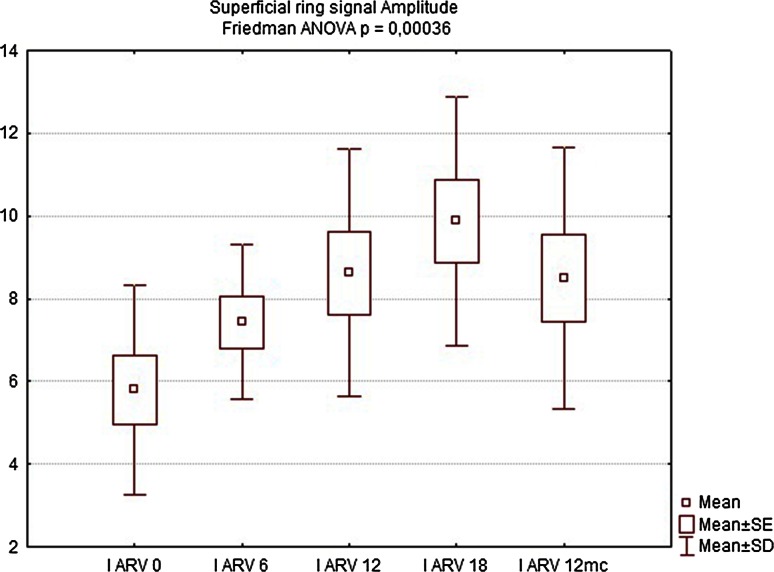
Electromyography signal amplitude (IARV) in µV initially and after 6, 12, 18 weeks and 12 months

**Fig. 11:**
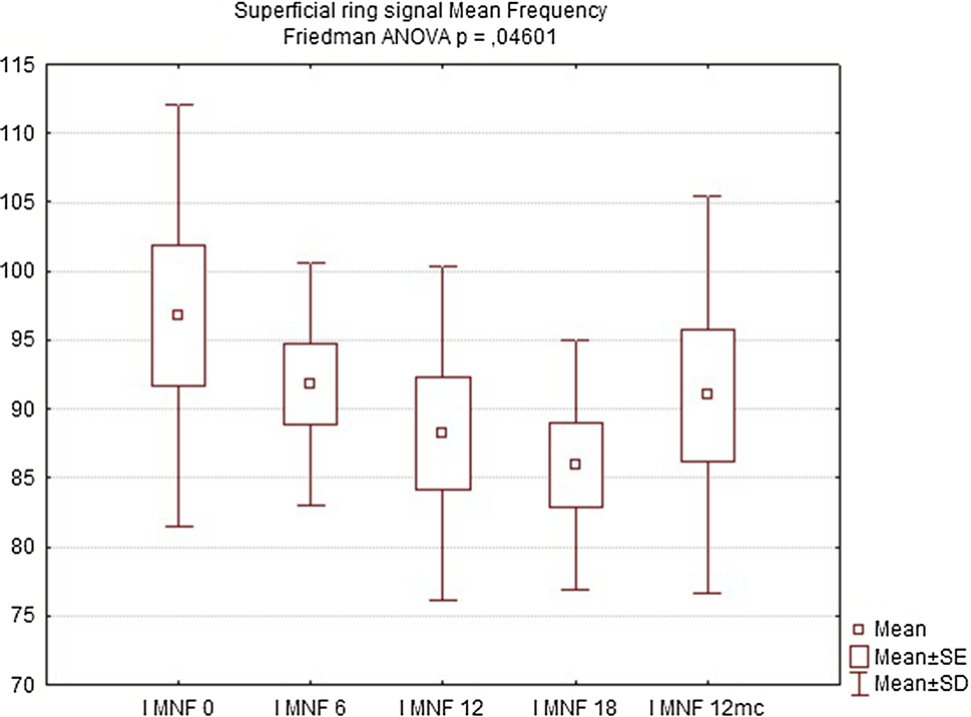
EMG signal frequency (IMNF) in Hz initially and after 6, 12, 18 weeks and 12 months

**Fig. 12:**
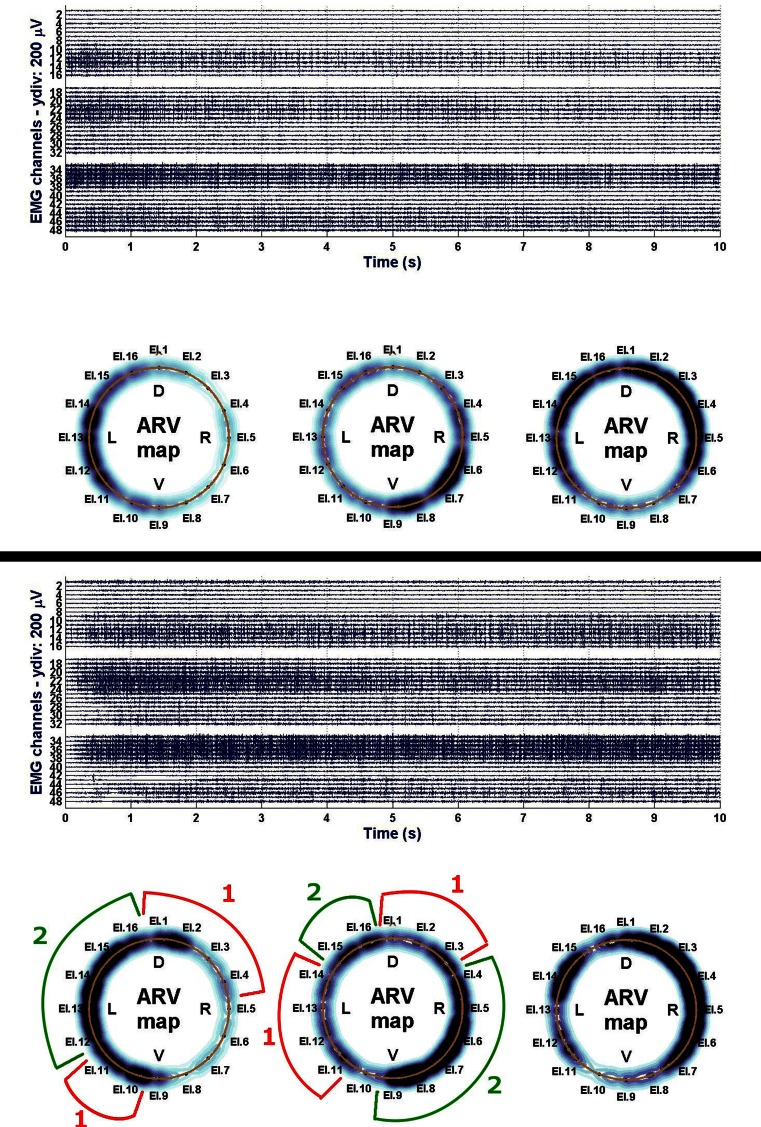
Example of electromyography power map—comparison of before (*top*) and 18 weeks after implantation (*bottom*). The darker the *blue color*, the stronger the electrical activity. Low activity on the *right* circumference of superficial part of the sphincter (*leftmost ring*) was due to external sphincter scarring and probable right-sided pudendopathy. Note electrical activity regained after implantation in previously inactive parts (*1*) and general increase in signal strength in undamaged muscle tissue (*2*)

EMG showed significant increase in signal amplitude (ARV) in all patients (Fig. 10), detecting an increased number of propagating action potentials (average increase by 60.3 % of the initial voltage value, Friedman ANOVA *p* < 0.05). There was also a significant change in mean or median frequency of the signal: Signal-to-noise ratio after implantation changed in favor of properly propagating motor unit action potentials (Fig. 11). Dimensional power map analysis also showed a general increase in signal amplitude (marked as “2” in Fig. 12). Moreover, in patients with signal gaps (scar tissue, without conductive or contractile abilities) action potentials appeared in previously inactive parts of the sphincter’s circumference (marked as “1” in Fig. 12).

**Fig. 13:**
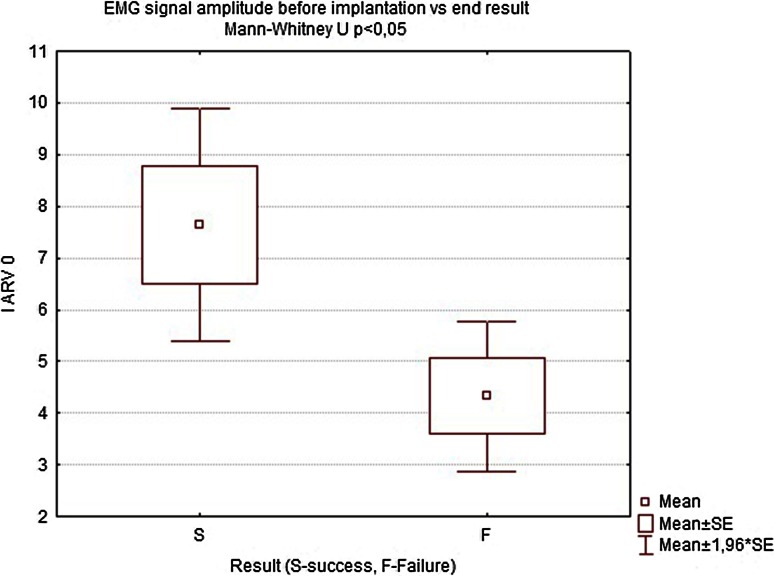
Comparison of electromyography signal amplitude (IARV) in Hz before implantation in patients who did and did not achieve satisfactory results

Twelve months after implantation, a deterioration of continence was reported by two of the six patients who achieved good results at 18 weeks. This was also reflected in the deterioration of manometric (BAP, SAP, HPZL) and EMG parameters (Figs. 5, 6, 7, 10, 11). Nevertheless, mean values were still significantly better than before the implantation. The remaining four patients (44.4 %) continued to have satisfactory results after 12 months, with reduction in frequency and intensity. Case-by-case analysis of the EMG signal showed that patients who achieved good end results had higher initial amplitude of action potentials (ARV measured before implantation) than those with poor or not persistent results (Mann–Whitney *U* test, *p* < 0.05) (Fig. 13).

## Discussion

Despite great interest in stem cells in general, there have been very few trials attempting to aid regenerative capabilities of human striated muscles. A damaged or weakened EAS is particularly difficult to strengthen without risky surgical intervention, and the only noninvasive method of EAS regeneration so far was biofeedback training, which, as shown by the literature, provided ambivalent results [7]. Implantation of autologous myoblasts may potentially not only improve general functioning of the EAS, but, by introduction of “fresh” muscle fibers, might increase the amount of contractile tissue in this region, thus improving the effect of various physiotherapeutic and behavioral treatments.

However, myocytes that differentiate from those stem cells require that the pelvic floor is sufficiently innervated to function properly, otherwise they would deteriorate with time [26]. In a pilot study by Frudinger et al. [24], autologous myoblasts were used in the treatment of anal incontinence due to obstetric anal sphincter injury, but the results evaluated by means of a questionnaire, manometric and ultrasound examinations were inconsistent and most strongly marked in subjective questionnaire assessment. In our study, patients also reported subjective improvement soon after implantation, even though no other test had shown any change yet, and there was no edema or other signs which might suggest a “bulking effect” of the injection itself (by the time of the 6-week visit, the suspension had been already completely absorbed). This led us to conclude that it is the placebo effect that might have been the key factor in the improvement of patients’ quality of life early after the procedure and questionnaires should be analyzed in the context of other tests. One of the methods of increasing the reliability of results that is used in many studies is anorectal manometry. There is strong supportive evidence in the literature for the use of anorectal manometry as a method of sphincter function assessment, and the importance of manometry assessment of colorectal surgery patients is only rarely contested [27–29]. Unfortunately, it gives no information about pudendopathy or other neurological conditions, potentially coexisting with sphincter damage. As for ERUS, it turned out to be even more subjective, as interpretation of results was very inconsistent and heavily depended on the opinion of the physician performing the examination. The only measurable variables in this examination, sphincter dimensions and sphincter scar dimensions, did not change significantly, and as a result, we excluded ultrasonography-related parameters from our analysis, as they did not provide any valuable information.

Taking all the above into consideration, all patients enrolled into our study group underwent surface endoanal EMG. The reliability of this noninvasive method of innervation assessment has been recently proved in several clinical and experimental studies [30–35]. The pelvic floor innervation of all the patients enrolled in our study was preserved, although to various degrees. The retrospective analysis of our data showed that the patients who had a weak response to the treatment or reported deterioration of initial positive effects had in fact low amplitude of motor units’ action potentials in initial examination, although the group was too small to show strong statistical correlation.

There are several limitations to our study. A small group of subjects is an obvious drawback. Moreover, there was no control group, because the limited number of patients who agreed to participate in the study made it impossible to perform a proper randomized controlled trial.

On the other hand, valuable result of this study was the EMG evidence of motor units’ action potentials gradually appearing in regions previously lacking of conductive and contractile capabilities, which may be a “proof of concept” that implanted myoblasts not only differentiated into myocytes, but were able to “plug-in” to existing motor units, with the result that individual motor neurons had more muscle tissue at their disposal.

## Conclusions

Implantation of autologous myoblasts gives good short-term results not only in a subjective assessment, but also in objective functional tests. Proper innervation of the EAS seems to be a crucial factor influencing final results. This promising technology can give a chance to improve the quality of life of patients with fecal incontinence, but it requires further study to achieve better and more persistent results, preferably in a controlled trial on larger groups of patients.
